# Anxious or Depressed and Still Happy?

**DOI:** 10.1371/journal.pone.0139912

**Published:** 2015-10-13

**Authors:** Philip Spinhoven, Bernet M. Elzinga, Erik Giltay, Brenda W. J. H. Penninx

**Affiliations:** 1 Institute of Psychology, Leiden University, Leiden, the Netherlands; 2 Department of Psychiatry, Leiden University Medical Center, Leiden, the Netherlands; 3 EMGO Institute for Health and Care Research, VU University Medical Center, Amsterdam, the Netherlands; Maastricht University, NETHERLANDS

## Abstract

This study aimed to examine cross-sectionally to what extent persons with higher symptom levels or a current or past emotional disorder report to be less happy than controls and to assess prospectively whether time-lagged measurements of extraversion and neuroticism predict future happiness independent of time-lagged measurements of emotional disorders or symptom severity. A sample of 2142 adults aged 18–65, consisting of healthy controls and persons with current or past emotional disorder according to DSM-IV criteria completed self-ratings for happiness and emotional well-being and symptom severity. Lagged measurements of personality, symptom severity and presence of anxiety and depressive disorder at T0 (year 0), T2 (year 2) and T4 (year 4) were used to predict happiness and emotional well-being at T6 (year 6) controlling for demographics. In particular persons with more depressive symptoms, major depressive disorder, social anxiety disorder and comorbid emotional disorders reported lower levels of happiness and emotional well-being. Depression symptom severity and to a lesser extent depressive disorder predicted future happiness and emotional well-being at T6. Extraversion and to a lesser extent neuroticism also consistently forecasted future happiness and emotional well-being independent of concurrent lagged measurements of emotional disorders and symptoms. A study limitation is that we only measured happiness and emotional well-being at T6 and our measures were confined to hedonistic well-being and did not include psychological and social well-being. In sum, consistent with the two continua model of emotional well-being and mental illness, a ‘happy’ personality characterized by high extraversion and to a lesser extent low neuroticism forecasts future happiness and emotional well-being independent of concurrently measured emotional disorders or symptom severity levels. Boosting positive emotionality may be an important treatment goal for persons personally inclined to lower levels of happiness.

## Introduction

“Mental illness is one of the main causes of unhappiness. This is not a tautology (…) for people can be unhappy for many reasons from poverty to unemployment to family breakdown to physical illness” [1]. According to the World Happiness Report 2103, self-reported mental illness is a highly influential and in affluent countries even the single biggest determinant of unhappiness [1]. The purpose of the present study is to critically examine the association between mental health and happiness while using structured interviews of psychiatric disorders. In addition, we aim to prospectively investigate whether 2, 4 and 6 years lagged values for personality characteristics such as neuroticism and extraversion are even more influential in predicting happiness over and above lagged values for common mental disorders.

The study of subjective well-being has grown dramatically in the last three decades with already more than 12,000 publications in 2012 [2]. In most studies the tripartite formulation of subjective well-being as originally proposed by Diener has been followed [3, 4]. According to this formulation subjective well-being is a multidimensional construct consisting of three separate components: (1) the presence of positive affect; (2) the relative lack of negative affect; and (3) people’s global evaluation of their life circumstances. This conceptualizations of subjective well-being differs along affective, temporal and cognitive dimensions [5]. Happiness and positive and negative affect are the affective components of subjective well-being. Happiness is the preponderance of positive affect over negative affect with a focus on an overall affective appraisal of life in general. On the other hand, general life satisfaction can be seen as a primarily cognitive evaluation of the quality of one's experiences, spanning an individual's entire life. Happiness and general life satisfaction refer to long-term states; positive and negative affect refer to the more recent occurrence of specific positive and negative emotions [5].

A large empirical literature shows that people who feel happier achieve better life outcomes, including financial success, supportive relationships, mental health, effective coping, and even physical health and longevity. Moreover, prospective and longitudinal studies show that happiness often precedes and predicts these positive outcomes rather than simply resulting from them [2–4, 6].

However, there is still some debate on the distinctiveness of mental health and mental illness and the issue of whether positive and negative emotions are relatively independent (the two continua view) or operate inversely (the one-dimensional view) [7]. According to the two continua model of mental illness and mental health, both concepts are related, but represent distinct dimensions. Several studies in the general population using different self-rated instruments for positive mental health and mental illness have provided support for the two continua model by showing that mental well-being and mental illness are inversely inter-related with correlations typically in the range of − 0.40 to − 0.60, indicating that measurements of positive mental health and mental illness belong to separable but correlated dimensions [8–11]. Interestingly these results parallel those of large-scale heritability studies on genetic and environmental influences on associations between subjective well-being and common mental disorders in adults [12–14]. These studies show that subjective well-being does not merely constitute the opposite end of a genetic liability to internalizing disorders, although some genetic factors for lower well-being convey risk for internalizing disorders. Higher levels of well-being also reflect other genetic influences associated with optimal psychological functioning.

Of note is that almost all available studies are general population studies, where psychological distress is generally assessed with self-report measures and not with formal DSM-IV psychiatric disorders. Only three community studies measuring formal DSM-IV psychiatric disorders showed that presence of psychopathology does not automatically imply absence of subjective well-being. An analysis of the cross-sectional Adult Psychiatric Morbidity Survey for England 2007 showed that many persons with common mental disorders still reported moderately high levels of emotional well-being [15]. Moreover, correlations of internalizing psychopathology [12] and major depressive disorder [14] with life satisfaction of around .60 have been reported for population-based samples of adult twins. Finally, in a pivotal clinical study Rapaport [16] assessed patients with affective or anxiety disorders who entered clinical trials. He observed that more persons with mood disorders (85% of persons with double/chronic depression, 63% with a current major depressive disorder and 56% with dysthymia) had life satisfaction scores outside 10% of the mean community normative value compared to persons with anxiety disorder (e.g., 21% of persons with social phobia and 20% of persons with panic disorder).

Besides psychopathology, personality traits may be one of the strongest personal determinants of happiness. In 1998 DeNeve and Cooper [5] performed a meta-analysis of the ‘happy personality’ based on 197 samples. The most prominent Big Five personality traits related to happiness specifically were extraversion (overall r = .27) and neuroticism (overall r = -.25). A more recent meta-analysis by Steel et al. [17], based on 347 samples, indicated even stronger links of personality with subjective well-being. Of the Big Five personality traits extraversion (overall r = .35) and neuroticism (overall r = -.30) were most strongly and consistently associated with happiness.

Crucially, neuroticism and extraversion are also the strongest of the Big Five personality traits associated with individual differences in psychopathology. A recent comprehensive review of the associations of higher order personality traits in the Big Three and Big Five models (i.e., neuroticism, extraversion, disinhibition, conscientiousness, agreeableness, and openness) and depressive, anxiety, and substance use disorders in adults, showed that all diagnostic groups scored high on neuroticism and low on conscientiousness. Many disorders were also associated with low levels of extraversion, with the largest effect sizes for dysthymic disorder and social anxiety disorder [18]. This poses the question whether the Big Five personality traits are differentially related to well-being and psychopathology. In the only study we are aware of, Lamers et al. [19] found in a representative community internet panel, that emotional stability (measured as reversed neuroticism) was differentially associated with self-reported psychopathology, whereas the personality traits of extraversion and agreeableness were uniquely associated with positive mental health.

To summarize, available evidence indicates that happiness and psychopathology constitute interrelated but distinct health dimensions and that neuroticism and extraversion may be differentially related to individual differences on both dimensions. To further advance our knowledge of happiness and psychopathology in relation to personality traits two main limitations must be addressed. First, almost all studies on the association of mental health with mental illness have been executed in community samples based on self-reported data for mental health. Second, as in most studies happiness and psychopathology have been measured concurrently, state-dependent perception and memory may constitute a common source of variance (i.e., levels of happiness may primarily reflect current mood) [20].

Therefore, the overarching goal of the present study was to examine cross-sectional as well as longitudinal relationships of happiness with both self-reported and clinician-rated anxiety and depression. More specifically our aims were: (a) to examine cross-sectionally whether persons with a current or past anxiety and/or depressive disorder as determined by a standardized, structured psychiatric interview report to be less happy than controls and whether level of anxiety and depression symptoms are related to happiness; and (b) to assess prospectively whether time-lagged measurements of personality traits of extraversion and neuroticism predict future happiness over and above the effect of time-lagged measurements of emotional disorders as well as symptom severity. We hypothesized that persons with past and current emotional disorders, in particular depressive disorders, would be less happy than controls and that symptom severity, in particular depression severity, would be related to happiness. Moreover, we expected that the personality traits of neuroticism and extraversion, would be predictive of future happiness independent of the presence of emotional disorders or level of symptom severity.

## Materials and Methods

### Study design

The Netherlands Study of Depression and Anxiety (NESDA) is an ongoing cohort study designed to investigate determinants, course and consequences of depressive and anxiety disorders. A sample of 2,981 persons aged 18 to 65 years was included, consisting of healthy controls, persons with a prior history of depressive and anxiety disorders, and persons with a current depressive and/or anxiety disorder. Respondents were recruited in the general population, through a screening procedure in general practice, or when newly enrolled in specialized health care in order to represent different health care settings and different developmental stages of psychopathology. General exclusion criteria were a primary diagnosis of severe psychiatric disorders such as psychotic, obsessive compulsive, bipolar or severe addiction disorder, and not being fluent in Dutch. A detailed description of the NESDA design and sampling procedures has been given elsewhere [21]. This study was approved by the Ethical Committees of VU University Medical Center, Leiden University Medical Center and University Medical Center Groningen and all respondents provided written informed consent prior to data collection.

The baseline assessment included demographic and personal characteristics, a standardized diagnostic psychiatric interview, an extensive set of psychological measures and a medical assessment including blood sampling. A face-to-face follow-up assessment was conducted after two (T2), four years (T4) and six years (T6), with a response of 87.1% (n = 2,596) at T2, 80.6% (n = 2,402) at T4, and 75.7% (n = 2,256) at T6. Happiness as our main outcome was only measured at T6 and consequently participants who answered the question on happiness (n = 2,142) constituted our present sample.

### Measures

#### Happiness and emotional well-being

Despite the large body of research on subjective well-being, it still remains unclear how the hypothesized three primary components [3, 4] of global life evaluation, positive affect and negative affect constitute, reflect, and/or combine to produce the construct of subjective well-being. As cogently put forward in an extensive and critical review of the tripartite structure of subjective well-being [22], the original proposition [3, 4] that described global life evaluation (happiness/life satisfaction), positive and negative affect as different aspects that should be assessed and examined separately to provide a full description of subjective well-being can be questioned. Ample empirical evidence shows that the associations between happiness, positive and negative affect are often substantial and robust and suggests that it is justified to derive latent factor or composite factor scores for subjective well-being as all three ingredients are required to assess subjective well-being completely [22]. Consequently, in the present study we choose to examine happiness both as an overall affective appraisal of one’s life and as subjective well-being in general (i.e. a composite of happiness and positive and negative affect).

#### Self-Rated Happiness (SRH)

In order to measure the degree of happiness at T6 we used the Self-Rating of Happiness scale (SRH) [23]. The SRH is a single-item self-rating scale consisting of the following question: "How happy or unhappy do you feel with your life in general?". Following this question a series of numbers from 1 to 7 with corresponding labels was written vertically: 1 ‘completely happy’; 2 ‘very happy’; 3 ‘quite happy’; 4 ‘neither happy, nor unhappy’; 5 ‘quite unhappy’; 6 ‘very unhappy’; and 7 ‘completely unhappy’. Measuring happiness by a single item is reliable, valid, and viable in community surveys as well as in cross-cultural comparisons [24]. The SRH was scored reversed, so that higher scores denote a higher degree of happiness.

#### Emotional Well-Being (EWB)

At T6 in addition to happiness, positive and negative affect was measured with the Mood and Anxiety Symptom Questionnaire—Shortened Dutch 30-item version (MASQ-D30) [25]. The MASQ-D30 is a validated short 30-item version of the longer 90-item MASQ [26] to measure Clark and Watson’s tripartite model (i.e. positive affect (PA), negative affect (NA) and somatic arousal (SA)). Individuals are asked to rate how much in the past week they have experienced “feelings, sensations, problems and experiences that people sometimes have” on a 5-point Likert scale, with 1 being “not at all” and 5 being “extremely”. The scales of the MASQ-D30 show good internal consistency and acceptable convergent validity [25]. We used the 10-item subscales for positive and negative affect for the purpose of the present study.

SRH, ‘MASQ: PA’ and ‘MASQ: NA’ scores showed large and significant (p < .001) inter-relations: SRH with PA (r = .62) and NA (-.64) and PA with NA (r = -.56). A principal component analysis of SRH, MASQ: PA and MASQ: NA (reversed scored, yielding ‘lack of negative effect’) scores yielded a clear one-factor solution with an Eigenvalue of 2.217 explaining 73.90% of the variance. The loadings were high: SRH = .88; MASQ: PA = .85; and MASQ: NA = -.85. We used the regression factor score as derived from this PCA as our composite index of subjective well-being and labeled this factor emotional well-being (EWB) throughout the rest of the text.

#### Psychiatric diagnosis

DSM-IV defined 6-month recency depressive (major depressive disorder, dysthymia) and anxiety (panic disorder with or without agoraphobia, social anxiety disorder, generalized anxiety disorder, agoraphobia without panic) disorders were assessed using the Composite Interview Diagnostic Instrument (CIDI, version 2.1) at T0, T2, T4, and T6. The CIDI is a fully standardized diagnostic interview, that is used worldwide to classify psychiatric diagnoses according to DSM-IV criteria [27]. It has shown high interrater reliability, high test-retest reliability and high validity for depressive and anxiety disorders [28]. The CIDI was administered in NESDA by more than 40 fully trained research assistants, including psychologists, nurses or residents in psychiatry.

#### Symptom severity

Severity of depression symptoms was measured with the 30-item Inventory of Depressive Symptomatology self-report version (IDS-SR) [29], which has shown high correlations with observer-rated scales such as the Hamilton Depression Scale [30]. Severity of generalized anxiety and panic symptoms was measured using the 21-item Beck Anxiety Inventory (BAI) [31]. This scale has shown sound psychometric properties such as factorial validity, internal consistency, and test-retest stability, as well as adequate convergent and discriminant validity [32].

#### Personality variables

The personality traits of neuroticism and extraversion were measured with the NEO Five-Factor Inventory (NEO-FFI) at T0, T2, and T4. The NEO-FFI is a 60-item questionnaire, which measures neuroticism and extraversion each on a 12-item subscale ranging from 12 to 60 per domain. Items are answered on a 5-point Likert scale, ranging from ‘strongly disagree’ to ‘strongly agree’ [33]. Internal consistency values range from .74 to .89 [33]. For the purpose of the present study we used the 12-item subscales for neuroticism and extraversion.

### Statistical analyses

First, differences in happiness (SRH) and emotional well-being (EWB) at T6 between the control, remitted, anxiety disorder only, depressive disorder only, and comorbid group were analyzed with ANOVA. Significant main effects for group were followed-up by post hoc Bonferroni comparisons.

To examine the cross-sectional relations of demographic characteristics (i.e., age, gender, and education) and each of the anxiety/depressive disorders (i.e., dysthymia, major depressive disorder, generalized anxiety disorder, social anxiety disorder, panic disorder, and agoraphobia) at T6 with SRH and EWB scores, we conducted two separate Structural Equation Models (SEM) with demographic and psychopathology variables as predictors and SRH or EWB scores as dependent variable. The advantage of this model is that it allows to examine the unique association of each of the disorders with SRH or EWB scores, while allowing comorbidity among disorders. The cross-sectional relationships of severity of anxiety (BAI) and depression (IDS) symptoms with SRH and EWB scores were examined with Pearson correlations as well as multiple regression analyses to study the unique relationship of IDS and BAI scores with SRH and EWB scores controlling for anxiety respectively depression symptoms and demographic variables.

To investigate the longitudinal association of depression (i.e., presence of major depressive disorder and/or dysthymia), anxiety (presence of at least one anxiety disorder) and personality characteristics (i.e., neuroticism and extraversion) with SRH and EWB scores, we conducted separate multiple regression analyses with SRH or EWB scores at T6 as dependent variable. In each analysis, we controlled for demographic variables (age, gender, and education). In addition time-lagged measurements for personality and presence of emotional disorders were entered in the prediction models, which were performed separately for the three different time points (T0, T2, and T4). We repeated these analyses using anxiety and depression symptom scores (instead of presence of anxiety and depressive disorder) at T0, T2 and T4 as independent variables. To guard against multicollinearity, the Variance Inflation Factor (VIF) score for each variable in each predictor model was examined. We used the arbitrary but stringent rules of thumb cut-off criterion of 2.5 for deciding when a given independent variable displayed “too much” multicollinearity.

We chose to report zero-order correlation (r) and semi-partial correlation (sr) coefficients, because correlation coefficients give a good indication of the size of the association and semi-partial correlation coefficients present the unique association of a particular variable with outcome over and above the effect of the other variables in the prediction model. In this way the attenuation of the zero-order correlations by controlling for all other variables in the model is easy to evaluate. We considered standardized estimates of less than .10 to represent a negligible, of ≥.10 to represent a small, of ≥.30 a moderate and of ≥.50 or larger a strong association. ANOVAs and multiple regression analyses were run using SPSS version 21 [34] and CFA and SEM models using MPlus v. 7.1 [35]. A significance level of p < .05 was used for all analyses.

## Results

### Sample characteristics

Of the 2256 persons at T6, 2142 (95.0%) completed the SRH, constituting the present study sample. Mean age was 48.2 years (SD = 13.1), mean number of years of education was 12.9 years (SD = 3.3) and 66.2% was female. 417 Persons had no previous history of depressive and/or anxiety disorder (control group), 1060 persons had a history of depressive and/or anxiety disorder but were disorder free at T6 (remitted group), and 597 persons had a current 6-month recency depressive and/or anxiety disorder at T6 (current group). Among these 597 participants, 330 persons had anxiety disorder only, 82 depressive disorder only, and 185 comorbid anxiety and depressive disorder. Psychiatric diagnoses among the current group were as follows: dysthymia: 157 (26.3%); major depressive disorder n = 145 (24.3%); generalized anxiety disorder n = 99 (16.6%); social anxiety disorder n = 333 (55.8%); panic disorder with/without agoraphobia n = 118 (19.8%); and agoraphobia without panic n = 170 (28.5%). The most frequent psychiatric comorbidity was social anxiety disorder with major depressive disorder (n = 100; 54.1% of the comorbid cases).

### Cross-sectional relations of happiness and emotional well-being with demographic characteristics, psychopathology status and symptom severity

SRH scores were significantly associated with age (r = -12, p < .001), while the association of EWB scores with age was not significant (r = -.02). Moreover, SRH and EWB scores were both significantly associated with education, even though the association was very small (r = .06, p < .01). Finally, females obtained significantly higher SRH scores (M = 5.05, SD = .97) than males (M = 4.88, SD = 1.08), t (2140) = 3.54, p < .001, but the EWB scores of females (M = -.17, SD = 4.7) did not differ from those of males (M = -.07, SD = 1.1, t (2140) = 5.91, ns).

**Fig 1:**
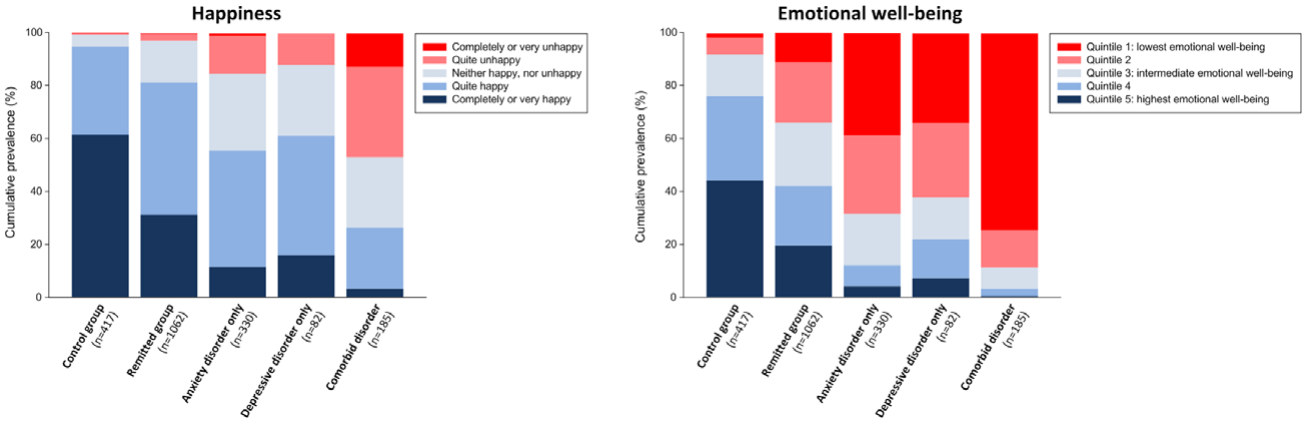
Histogram of happiness and emotional well-being scores across groups differing in psychopathology in 2142 participants from the Netherlands Study of Depression and Anxiety (NESDA).

Next, using ANOVA we compared SRH and EWB scores between psychopathology groups (control, remitted, anxiety disorder only, depressive disorder only, and comorbid group). As can be derived from Table 1, a significant effect for group was found for each of the dependent variables (all p < .001). Subsequent post hoc Bonferroni comparisons, showed that controls had significantly higher SRH and EWB scores than persons with a previous anxiety/depressive disorder, who obtained significantly higher SRH and EWB scores than persons with anxiety disorder only and depressive disorder only of which the scores did not differ significantly. Persons with comorbid disorder had significantly lower SRH and EWB scores than persons with anxiety only and persons with depressive disorder only. The proportion of persons answering on the SRH that they were ‘quite unhappy’, ‘very unhappy’ or ‘completely unhappy’ across groups was as follows: control: 0.7%; remitted: 3.0%; anxiety only: 15.5%; depression only: 12.2% and comorbid group: 47.0% (see Fig 1). Repeating the ANOVA’s with age, gender and education as covariates yielded similar results (data not shown).

**Table 1:** Happiness and emotional well-being scores in groups differing in psychopathology (n = 2076) ^a^.

	1. Controls (n = 417)		2. Past disorder (n = 1060)		3. Anxiety disorder only (n = 330)		4. Depressive disorder only (n = 82)		5. Comorbid disorder (n = 185)		F (4,2071)	η_p_^2^	Post hoc contrasts
Variable	M	SD	M	SD	M	SD	M	SD	M	SD			
Happiness	5.64	.75	5.12	.83	4.50	.94	4.65	.89	3.69	1.10	200.31 *	.28	1>2>3 = 4>5
EWB	.75	.61	.16	.80	-.60	.89	-.46	.92	-1.42	.95	302.70 *	.37	1>2>3 = 4>5

Note.

^a^ = Missing data about past disorder between T0 and T6 for 66 persons;

EWB = Composite regression factor score for emotional well-being based on happiness and positive and negative (reversely scored) affect scores;

* = p < .001.

IDS scores showed a significant and large relationship with SRH (r = .66, p < .001) and EWB scores (r = .80, p < .001). BAI scores had a significant and moderately large relationship with SRH (r = .49, p < .001) and large relationship with EWB scores (r = .63, p < .001). Controlling for anxiety severity and demographic characteristics, the association of IDS scores with SRH scores was attenuated and became .45 (p < .001) and .50 (p < .001) with EWB scores. Controlling for depression severity and demographic characteristics, the association of BAI scores with SRH scores was greatly attenuated and became negligible .06 (p < .01) and with EWB scores -.01 (ns).

### Cross-sectional relations of happiness and emotional well-being with individual psychiatric diagnoses

**Fig 2:**
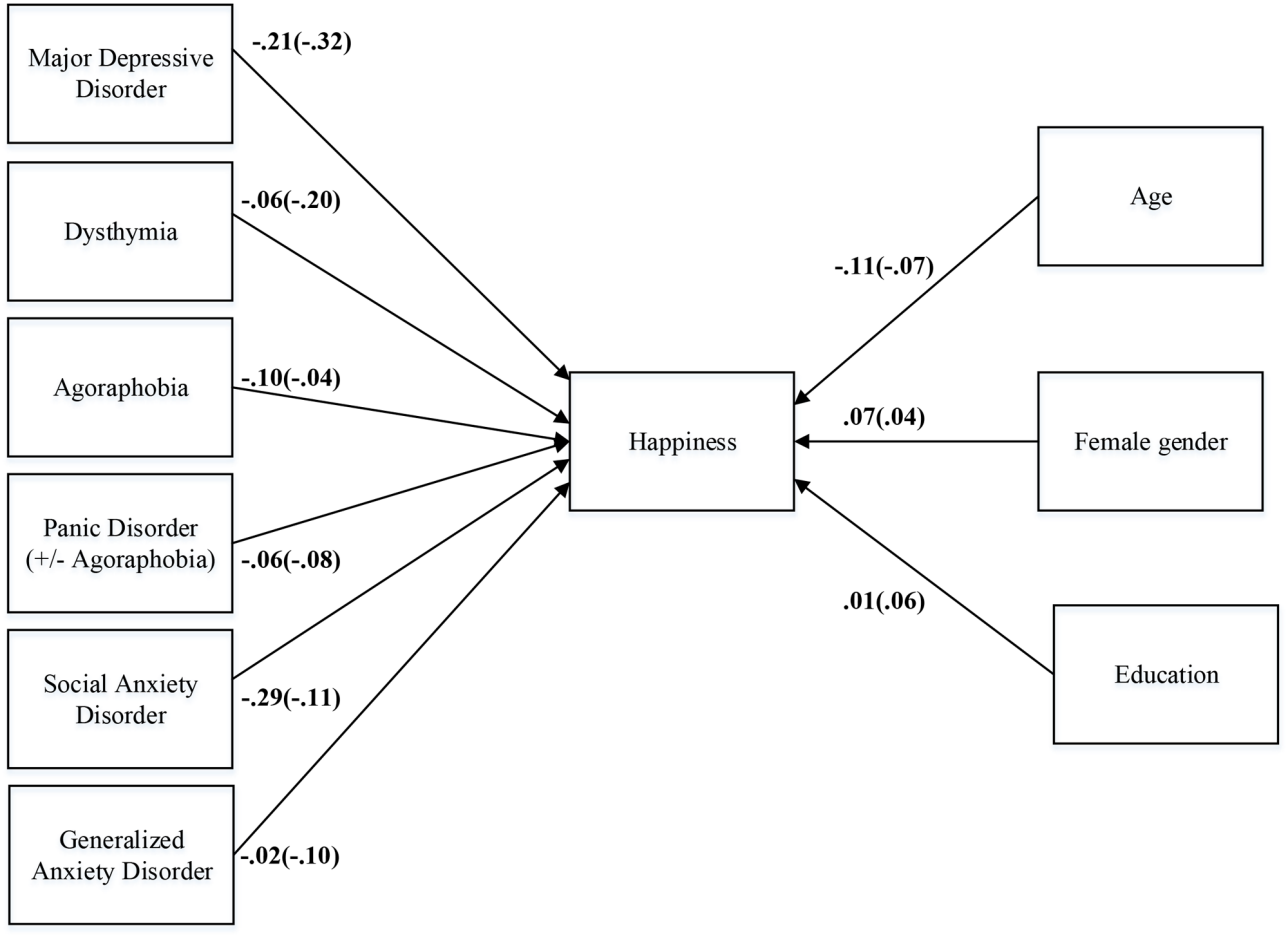
Model of the relation of demographic and psychopathology variables with happiness and emotional well-being. Parameter estimates for the model of the direct relationship of different depressive and anxiety disorders (dummy coded as ‘1’) with happiness and emotional well-being in 2142 participants from the Netherlands Study of Depression and Anxiety (NESDA) controlling for age, gender and education. Single-headed arrow path coefficients represent fully standardized semi-partial regression coefficients. Estimates within parentheses represent the regression coefficients for the relation of demographic and psychopathology variables with emotional well-being. All estimates are statistically significant at p < .05 (except the paths from education and generalized anxiety disorder to happiness).

Applying Structural Equation Modeling (SEM), we conducted a saturated model in which SRH or EWB scores were predicted by demographic characteristics and each of the psychiatric diagnoses (see Fig 2). As can be derived from this model, which by definition has a perfect fit, depressive disorders and to a smaller extent social anxiety disorder showed the largest unique associations with both SRH and EWB scores. The associations of SRH scores with education and generalized anxiety disorder were non-significant.

### Longitudinal relations of emotional disorders and personality variables with happiness and emotional well-being

Next, we executed multiple regression analyses with SRH or EWB scores at T6 as dependent variable. In each analysis demographics (i.e., age, gender, and education) were entered as control variables. Time-lagged depressive and anxiety, disorder and personality characteristics (i.e., neuroticism and extraversion) were entered as additional predictor variables. We performed separate analyses for T0, T2, and T4 assessments. None of the independent variables displayed “too much” multicollinearity (range of VIF values: 1.01–1.85). Personality traits, emotional disorders and symptom severity proved to be stable over time: neuroticism scores (T0 with T2: r = .79; T2 with T4: r = .80); extraversion scores (T0 with T2: r = .81; T2 with T4: r = .83); presence of a depressive disorder (T0 with T2: r = .35; T2 with T4: r = .39); presence of a anxiety disorder (T0 with T2: r = .43; T2 with T4: r = .41); depression severity scores (T0 with T2: r = .73; T2 with T4: r = .77); anxiety severity scores (T0 with T2: r = .69; T2 with T4: r = .74).

As can be derived from Model 1 in Table 2, the correlation of demographic, disorder and personality variables simultaneously with SRH scores was large (T0: r = .52; T2: r = .54; and T4: r = .58). Moreover, the moderately large zero-order correlations of depressive and anxiety disorder and personality variables at T0, T2 and T4 with SRH scores at T6 were greatly attenuated by controlling for all the other demographic, disorder and personality variables. Of the disorder variables only the most proximal semi-partial correlation of depressive disorder at T4 with SRH scores at T6 was statistically significant with a small effect size of at least .10 (T4: sr = -.15). Depressive and anxiety disorders at other time points showed non-significant or significant but negligible associations (smaller than .10) with SRH at T6. Of the personality variables, both neuroticism (T0: sr = -.19; T2: sr = -.18; T4: sr = -.19) and extraversion (T0: sr = .18; T2: sr = .19; and T4: sr = .18) showed small and significant semi-partial correlations with SRH scores across all time points.

**Table 2:** Zero-order and semi-partial correlation coefficients of demographic, psychopathology and personality characteristics at T0, T2, T4, and T6 with happiness assessed at T6.

Variables	T0		T2		T4	
	r	sr	r	sr	r	sr
*Model 1*: *Disorders and personality*						
Dep. Disorder	-.30 ***	-.07 ***	-.31 ***	-.07 ***	-.37 ***	-.15 ***
Anx. Disorder	-.22 ***	-.02	-.28 ***	-.04	-.28 ***	-.03
Neuroticism	-.43 ***	-.19 ***	-.46 ***	-.18 ***	-.49 ***	-.19 ***
Extraversion	.45 ***	.18 ***	.47 ***	.19 ***	.49 ***	.18 ***
*All variables*	.52 ***		.54 ***		.58 ***	
*Model 2*: *Symptom Severity and personality*						
Dep. Severity	-.47 ***	-.14 ***	-.51 ***	-.16 ***	-.55 ***	-.20 ***
Anx. Severity	-.34 ***	.03	-.39 ***	.02	-.41 ***	.02
Neuroticism	-.43 ***	-.08 ***	-.46 ***	-.08 ***	-.49 ***	-.09 ***
Extraversion	.45 ***	.16 ***	.47 ***	.16 ***	.49 ***	.15 ***
*All variables*	.54 ***		.57 ***		.60 ***	

r = zero-order correlation coefficient; sr = semipartial correlation coefficient; Dep. Disorder = Depressive disorder (i.e., dysthymia, major depressive disorder); Anx. Disorder = Anxiety disorder (i.e., social anxiety disorder, generalized anxiety disorder, panic disorder with/without agoraphobia; agoraphobia); Dep. Severity = Inventory of Depressive Symptomatology (IDS) score; Anx. Severity = Beck Anxiety Inventory (BAI) score;

*** p < .001;

** p < .01;

* p < .05.

Repeating these multiple regression analyses with EWB scores as dependent variable essentially yielded similar results (see Model 1 in Table 3). As to be expected the correlation of all variables simultaneously with EWB scores was somewhat higher (T0: r = .60; T2: r = .65; and T4: r = .67) as EWB is a composite dependent variable based on ‘MASQ; PA’ and ‘MASQ: NA’ scores in addition to SRH scores. The moderately large zero-order correlations of depressive and anxiety disorder and large zero-order correlations of personality variables at T0, T2 and T4 with EWB scores at T6 were greatly attenuated by controlling for all the other demographic, disorder and personality variables. Again of the disorder variables only the most proximal semi-partial correlation of depressive disorder at T4 with SRH scores at T6 was statistically significant with a small effect size of at least .10 (T4: sr = -.12). Neuroticism (T0: sr = -.24; T2: sr = -.24; T4: sr = -.25) and extraversion (T0: sr = .19; T2: sr = .20; and T4: sr = .19), however, showed small and significant semi-partial correlations with EWB scores across all time points.

**Table 3:** Zero-order and semi-partial correlation coefficients of demographic, psychopathology and personality characteristics at T0, T2, T4, and T6 with emotional well- being as assessed at T6.

Variables	T0		T2		T4	
	r	sr	r	sr	r	sr
*Model 1*: *Disorders and personality*						
Dep. Disorder	-.35 ***	-.06 ***	-.36 ***	-.06 ***	-.40 ***	-.12 ***
Anx. Disorder	-.30 ***	-.01	-.35 ***	-.01	-.35 ***	-.05 **
Neuroticism	-.54 ***	-.24 ***	-.58 ***	-.24 ***	-.60 ***	-.25 ***
Extraversion	.51 ***	.19 ***	.54 ***	.20 ***	.56 ***	.19 ***
*All variables*	.60 ***		.65 ***		.67 ***	
*Model 2*: *Symptom Severity and personality*						
Dep. Severity	-.55 ***	-.14 ***	-.61 ***	-.15 ***	-.64 ***	-.18 ***
Anx. Severity	-.42 ***	.01	-.50 ***	-.01	-.51 ***	-.02
Neuroticism	-.54 ***	-.13 ***	-.58 ***	-.12 ***	-.60 ***	-.14 ***
Extraversion	.51 ***	.16 ***	.54 ***	.17 ***	.56 ***	.16 ***
*All variables*	.62 ***		.67 ***		.70 ***	

r = zero-order correlation coefficient; sr = semipartial correlation coefficient; Dep. Disorder = Depressive disorder (i.e., dysthymia, major depressive disorder); Anx. Disorder = Anxiety disorder (i.e., social anxiety disorder, generalized anxiety disorder, panic disorder with/without agoraphobia; agoraphobia); Dep. Severity = Inventory of Depressive Symptomatology (IDS) score; Anx. Severity = Beck Anxiety Inventory (BAI) score;

*** p < .001;

** p < .01;

* p < .05.

As a final step we repeated the analyses described above with anxiety and depression symptom severity as predictor variables (see Model 2 in Tables 2 and 3). VIF values for depression and anxiety symptom severity and neuroticism were above the cut-off of 2.5 varying from 2.46 to 4.01 indicating that the standard error for the coefficient of these predictor variables is maximally two times as large as it would be if that predictor variable were uncorrelated with the other predictor variables. These analyses yielded similar results with correlations of all variables simultaneously with SRH scores varying from .54 to .60 and with EWB scores ranging from .62 to .70. Again extraversion showed small and significant semi-partial correlations with SRH scores (range of semi-partial correlations: .15 to .16) and EWB scores (range of semi-partial correlation: .16 to .17) across all time points. However, the association of depression symptom severity with SRH scores (range of semi-partial correlations: -.14 to -.20) and EWB scores (range of semi-partial correlations: -.14 to -.18) was significant and small across all time points, while the association of neuroticism with SRH scores remained significant but became negligible (range of semi-partial correlations: -.08 to -.09) and with EWB scores remained significant but became smaller in size (range of semi-partial correlations: -.12 to -.14)

## Discussion

The first aim of the present study was to examine cross-sectionally whether persons with anxiety or depression as determined by self-report or a standardized psychiatric interview report to feel less happy in general or manifest less emotional well-being than controls. As hypothesized we found that the overall affective appraisal of being happy and the level of emotional well-being gradually decreased from controls to persons with a past anxiety/depressive disorder, to persons with a current single anxiety or single depressive disorder to persons with a current comorbid anxiety/depressive disorder. Examining the association of happiness and emotional well-being with specific anxiety and depressive diagnoses into more detail showed that happiness and emotional well-being were most strongly related to depressive disorders and contrary to our expectation also to social anxiety disorder. Relationships of happiness and emotional well-being with generalized anxiety disorder, panic disorder and agoraphobia were much smaller. In line, severity of self-reported depression but not anxiety symptoms showed a unique relationship with level of happiness and emotional well-being. The level of happiness of controls and persons with remitted disorder seemed comparable to the level of happiness as found in Dutch general population surveys in which less than 5% of the respondents give an insufficient score for happiness of 5 or less on a 0–10 scale. However, a much larger proportion of persons with a current single disorder (12–16%) and in particular comorbid disorder (47%) indicated to feel unhappy to a certain extent in the present study. Thus, while the large majority with a current single depressive or anxiety disorder indicates to be still reasonably happy, almost half of the persons with psychiatric comorbidity feel unhappy to a certain extent.

Our results only partly concur with those of Rapaport [16], who observed more severely impaired general life satisfaction in depressive compared to anxiety disorders. In our study happiness and well-being were not only associated with current major depressive disorder, but also with current social anxiety disorder. These results are consistent with numerous studies showing the importance of diminished positive psychological experiences in excessive social anxiety [36] and suggest that as in major depressive disorder a lack of positive affect may also constitute a disorder-specific characteristic underlying impaired subjective well-being in social anxiety disorder. Another study difference relates to the higher degree of impairment in well-being, as in the study of Rapaport 65 to 85% of the participants with depressive disorders reported impaired life satisfaction in the severely impaired range. These differences in study outcome may be primarily due to sample differences (patients selected for medication trials versus unselected persons from the community, primary and specialized mental health care). Moreover, in interpreting the relatively low percentage of unhappy participants in the present study also cultural factors have to be taken into account, as the Netherlands belong to the five most happiest countries in the world together with Denmark, Norway, Switzerland, and Sweden [37].

Although we primarily used demographic variables (i.e., age, gender, education) as control variables, it is worth noting that as in previous studies demographic variables only accounted for a small proportion in the variance of happiness or subjective well-being scores, which was one of the reasons that the scope of research into subjective well-being has broadened to include personality traits [3]. A review of the research literature on the relation of demographic variables with subjective well-being [4] showed no consistent relation of age or gender with subjective well-being. However, a consistent positive association of level of education with subjective well-being has been found across many studies.

Our second study aim was to assess prospectively whether time-lagged measurements of the personality traits of neuroticism and extraversion and concurrently measured emotional disorders or symptom levels predict future happiness and emotional well-being. Our findings show that extraversion is positively and neuroticism is inversely related with happiness and emotional well-being with moderate to large correlations. However, in multivariable analyses these zero-order correlations were greatly attenuated. Extraversion showed a significant and small relationship with happiness and emotional well-being after controlling for demographic, emotional disorder or symptom severity variables and neuroticism across all measurement moments. Controlling for demographic, emotional disorders variables and extraversion also neuroticism showed a significant and small relationship with happiness and emotional well-being across all time points, but controlling for symptom severity these relationships became negligible in size. These results suggest that of the personality factors in particular extraversion uniquely contributes to the overall affective appraisal of being happy and emotional well-being over and above the effect of emotional disorders or symptom severity as to be expected on the basis of the two continua model of emotional well-being and mental illness. Our results extend those of numerous cross-sectional studies of the relation of neuroticism and extraversion with happiness in normal samples [5, 17] by showing that even 6-year lagged values for these personality traits predict future happiness and emotional well-being, also in the presence of emotional disorders and taking symptom severity into account.

In accordance with the cross-sectional analyses, depressive and anxiety disorders showed small to moderately large zero-order associations with future happiness and emotional well-being. In line depression and anxiety symptom severity showed moderately large to large zero-order associations with future happiness and emotional well-being. In multivariable analyses these zero-order correlations were greatly attenuated. Depression symptom severity showed a significant and small relationship with happiness and emotional well-being after controlling for demographic and personality variables and anxiety symptom severity across all measurement moments. Moreover, the 2-year lagged value of depressive disorder had a small effect on happiness and emotional well-being. These results suggest that the predictive value of depressive disorder for future happiness and well-being may be more time-and state-dependent than the predictive value of depressions symptom severity. Apparently, quite stable levels of depression symptoms that are intrinsically intertwined with neuroticism are more influential in determining future happiness and emotional well-being than more discrete episodes of a depressive disorder.

A first limitation of the present study is that we only measured happiness and not general life satisfaction as a more cognitive evaluation of the quality of one's life experiences. Moreover, we only measured subjective well-being from a hedonistic perspective. Partly because of discontent with this narrow definition [38,39], another research tradition focuses on meaning and self-realization and defines well-being in terms of the degree to which a person is optimally functioning [39,40]. In this so-called eudaimonic approach, individuals are psychologically healthy when they are fully functioning in life, as manifested by self-acceptance, personal growth, autonomy, purpose in life, a sense of mastery, and positive relations with important others [38]. More recently, a third approach also following the eudaimonic tradition emphasizes social well-being and states that effective functioning can only be fully understood when optimal functioning in community life is included [41]. Separate lines of research have investigated hedonic, psychological and social well-being [40], but the few studies which investigated differences and similarities between these concepts of well-being showed a significant overlap [11, 39, 42].

A recent study even showed that a latent factor consisting of the above dimensions of well-being perfectly fitted the data and that a latent general factor of well-being showed a high continuity from age 36 to 42 (standardized coefficient 0.84) [43]. These results are in concordance with our study findings showing that cross-sectional and longitudinal associations of demographic, psychopathology and personality variables with happiness were comparable to those with a composite score of emotional well-being also based on positive affect and negative affect. By using two indices for subjective well-being, we were able to show that associations between global affective evaluation of life, positive and negative affect are substantial and that it seems justified to derive composite factor scores for subjective well-being as all three ingredients may be required to assess subjective well-being [22].

A second limitation of our study is that we only measured SRH and EWB at one wave of our study. Available studies suggest that subjective well-being scores are quite stable over time [44]. For example, in a panel of respondents followed for many years even the one-year stability coefficient for happiness assessed with a single item was about .56 [45]. This stability is to be expected as subjective well-being critically depends on the stable personality traits of neuroticism and extraversion [5, 17]. In addition, several genetic studies also provide support for the stability of subjective well-being showing that genetic influences account for as much as 35–50% of the phenotypic variance in life satisfaction [46–54]. Moreover, the long-term stability of subjective well-being across intervals of 6 and 10 years was mainly attributable to stable genetic factors [48,54]. Finally, the same genetic information responsible for individual variation in neuroticism and extraversion has been shown to explain individual variations in subjective well-being as most of the common additive and non-additive genetic variance in subjective well-being is shared with neuroticism and extraversion [55,56].

On the basis of these results one would expect that personality traits and subjective well-being will show a consistent and positive association over time. This hypothesis was evaluated in a recent Finnish longitudinal cohort study examining associations of personality traits with life satisfaction across ages 33–50 with bivariate latent growth curve analyses. Results indicated that a low initial level of neuroticism (.44) and high level of extraversion (.20) correlated strongly with a high initial level of life satisfaction. Interestingly, initial levels of personality traits did not predict changes in life satisfaction suggesting that stable levels of personality traits contribute to life satisfaction in general [57].

The large determination of subjective well-being by genetic and personality factors does not have to be a reason for therapeutic pessimism. Firstly, a recent genetic study showed that life satisfaction also showed substantial non-shared environmental influences independent of personality traits suggesting that environmental influences per se can make people more satisfied with life [56]. Secondly, in discussing mechanisms explaining the observed linkages between personality and subjective well-being, Steel et al. [17] mention among others a “nature via nurture” perspective according to which this relation is indirect and partly mediated by environmental factors. As individuals who possess low levels of extraversion or high levels of neuroticism are less likely to position themselves in positive life situations [58], boosting positive emotionality may be an important treatment goal. Positive psychology interventions primarily aimed at raising positive feelings, positive cognitions or positive behavior as opposed to interventions aiming to reduce symptoms, problems or disorders have been shown to be effective in enhancing subjective and psychological well-being [59,60]. These interventions may be especially indicated for persons who perhaps are not “born to be happy” [46], but can in this way compensate for their natural shortcomings.

In conclusion, the present study showed that happiness and emotional well-being are affected by the presence of an emotional disorder and higher levels of symptom severity. More specifically, persons with more severe depression symptoms, persons with a depressive or social anxiety disorder and in particular persons with comorbid emotional disorders report lower levels of happiness and emotional well-being. In accordance with the two continua model of emotional well-being and mental illness, a ‘happy’ personality characterized by high extraversion and to a lesser extent low neuroticism forecasts future happiness and emotional well-being after 2, 4 or 6 years independent of concurrently measured emotional disorders or symptom severity levels. Boosting positive emotionality may be an important treatment goal for persons personally inclined to lower levels of happiness.
